# Possible and Probable Dementia and Depressive/Anxiety Symptoms in U.S. Older Adults: One-Year Follow-Up

**DOI:** 10.1177/08982643251369347

**Published:** 2025-08-20

**Authors:** Namkee G. Choi, Yuanjin Zhou, C. Nathan Marti

**Affiliations:** 1 School of Social Work, 12330 The University of Texas at Austin, Austin, TX, USA

**Keywords:** depression, anxiety, possible dementia, probable dementia, bidirectional association

## Abstract

Using data from the 2022 and 2023 U.S. National Aging and Health Trends Studies (*N* = 4,942 sample persons interviewed in both years), we examined cross-sectional and one-year lagged bidirectional relationships between the severity of cognitive impairment and depressive/anxiety symptoms. We fitted multinomial logistic and linear regression models for cross-sectional associations and a path model for one-year cross-lagged associations. Depressive/anxiety symptoms were cross-sectionally associated with a higher likelihood of both possible (mild) and probable (advanced) dementia compared to no dementia, with an even greater likelihood for probable dementia. Depressive/anxiety symptoms in 2022 were significantly associated with 2023 probable dementia, and probable dementia in 2022 was significantly associated with depressive/anxiety symptoms in 2023. The findings underscore the importance of early identification and concurrent management of both cognitive decline and depressive/anxiety symptoms in older adults.

## Introduction

A plethora of research has shown that the majority of older adults with dementia, with Alzheimer’s Disease (AD) as the most common type, experience neuropsychiatric symptoms, including depression and anxiety, during the disease process (Fisher et al., 2024). Alongside the cross-sectional associations between elevated depressive symptoms and poorer cognitive functioning (e.g., memory, orientation, verbal fluency, and executive function), multiple longitudinal studies have found that worsening depressive symptoms and anxiety over time, as well as long-term persistent depressive symptoms/anxiety, are linked to an accelerated decline in cognitive function (Ahn et al., 2021; Formánek et al., 2020; Gulpers et al., 2016; Lin et al., 2025; Santabárbara et al., 2019, 2020; Yang et al., 2020; Yin et al., 2024; Zheng et al., 2018; Zhu et al., 2023). Since affective and emotional dysregulation is common in preclinical and prodromal dementia syndromes, often acting as a harbinger of neurodegenerative change and progressive cognitive decline (Ismail et al., 2018), late-life depression/anxiety is considered a risk factor for dementia and a prodrome for a subsequent neurodegenerative process over time.

Research has also suggested bidirectional associations between depression/anxiety and cognitive decline. For instance, in a clinical-pathologic cohort study spanning nearly 8 years, Wilson et al. (2014) found that incident dementia was associated with higher levels of depressive symptoms before the onset of dementia, and these symptoms then deteriorated more rapidly after dementia began. Botto et al.‘s systematic review (2022) concluded that depression/anxiety in dementia, particularly AD, can be attributed to the neurodegeneration of areas and circuits responsible for emotional processing. The authors further noted that dysthymia (i.e., persistent depressive disorder with mild-to-moderate chronic depression) arises in the early stages of AD as an emotional response to the awareness of progressive cognitive decline and the loss of functional abilities and can also contribute to that decline, and that anxiety may initially manifest as a compensatory behavior.

Compared to the well-established evidence of the longer-term associations between worsening or persistent depressive/anxiety symptoms and cognitive decline and dementia, relatively little research has been done on the contemporaneous and bidirectional associations between cognitive decline and depressive/anxiety symptoms. An earlier systematic review found consistent cross-sectional associations between subjective cognitive impairment and affective symptoms, suggesting that depressive/anxiety symptoms may arise in response to awareness of dementia, likely driven by fears of loss of function (Hill et al., 2016). A recent population-based cohort study indicated that subjective cognitive impairment preceded depressive symptoms during the development of cognitive decline and dementia, with high levels of depressive symptoms linked to an increased risk of dementia conversion in individuals with subjective cognitive decline (Kleineidam et al., 2023). Qualitative studies of older adults’ reactions to presumptive subjective cognitive decline or memory concerns also show their distress and worry about losing independence, social roles, and self-identity (Carter et al., 2025; Kinzer & Suhr, 2016; Maxfield et al., 2024). Healthy late middle-aged and older adults expressed fear of a future diagnosis of AD or other dementia and anticipated suicidal thoughts or death ideation if diagnosed with dementia (Maxfield et al., 2023, 2024).

Although these studies of subjective cognitive impairment are informative, more population-based research is warranted to elucidate the contemporaneous as well as short-term bidirectional relationships between validated indicators of cognitive decline and depressive/anxiety symptoms. Emotional responses to cognitive deterioration, such as distress, sadness, and fear of losing autonomy or self-identity, may progress to clinically significant psychiatric symptoms, further compromising quality of life and exacerbating functional impairment. Conversely, depression/anxiety may impair attention, memory, and executive functioning, thereby hastening cognitive decline and possibly contributing to neurodegenerative processes. Examining these short-term reciprocal associations is critical for improving clinical care for older adults with dementia and/or depressive/anxiety symptoms, advancing understanding of the affective consequences and antecedents of cognitive decline, and informing the development of timely, targeted interventions and preventive strategies.

In the present study, based on two consecutive National Health and Aging Trends Study (NHATS) interview waves (2022 [T1] and 2023 [T2]) of a panel of U.S. Medicare beneficiaries, we first examined T2 cross-sectional bidirectional relationships between the severity of cognitive impairment and depressive/anxiety symptoms. We then examined one-year cross-lagged associations between T1 depressive/anxiety symptoms and T2 dementia status as well as between T1 dementia status and T2 depressive/anxiety symptoms, controlling for T1 dementia status, T1 depressive/anxiety symptoms, and sociodemographic, health, and social support characteristics. Following the NHATS dementia classification, the severity of cognitive impairment was denoted as possible (mild) versus probable (advanced) dementia (described in more detail in the Measures section). Since depression and anxiety frequently coincide, with highly correlated symptoms, and act as mutual risk factors, regardless of age groups, resulting in comorbid depression and anxiety (Bian et al., 2024; Jacobson & Newman, 2017; King-Kallimanis et al., 2009), we included depressive/anxiety symptoms as assessed in the NHATS.

Based on previous study findings, the study hypotheses were: (H1) Both possible and probable dementia would be significantly associated with depressive/anxiety symptoms and vice versa cross-sectionally; however, the association would be stronger for probable dementia; (H2) T1 depressive/anxiety symptoms would be significantly associated with T2 possible and probable dementia, with stronger associations for probable dementia; and (H3) T1 possible and probable dementia would be significantly associated with T2 depressive/anxiety symptoms, with stronger associations for probable dementia. Covariates included sociodemographic, health, and social support characteristics that are known risk factors for both dementia and depressive/anxiety symptoms (Livingston et al., 2024; Vink et al., 2008). By controlling for these covariates, we examined independent associations between depressive/anxiety symptoms and possible or probable dementia. The study findings contribute to the existing knowledge base about the short-term bidirectional association between cognitive impairment and depressive/anxiety symptoms.

## Methods

### Data and Sample

Starting in 2011, the NHATS has collected data annually from a nationally representative panel of Medicare beneficiaries ages 65 and older concerning their physical, functional, cognitive, and sensory capabilities; social, physical, and technological environments; and participation in valued activities (https://www.nhats.org/). The 2022 and 2023 data were collected through in-person interviews, with the 2023 interviews including the NHATS third replenishment samples. In this study, we focused on the sample persons interviewed in both 2022 and 2023 (*N* = 4,942) who lived in their own homes or residential care communities and self-reported data in both years to examine both cross-sectional and one-year lagged associations between dementia and depressive/anxiety symptoms. Our decision to exclude 202 proxy-interviewed older adults, most of whom (*n* = 132) had more advanced dementia, was based on previous study findings that emotional responses and their expression tend to be reduced in severe cognitive impairment or late AD stages (Botto et al., 2022). This study, based on the analysis of de-identified public-use data, was exempt from review by the authors’ institutional review board.

### Measures

*Dementia status (no dementia, possible dementia, and probable dementia) in 2022 and 2023* was determined using the most recently updated NHATS dementia classification algorithm based on two types of information for self-respondents: (1) doctor diagnosis of dementia or AD (yes or no) regardless of cognitive test results; and (2) scores from cognitive tests evaluating the sample person in the following three domains: memory (immediate and delayed 10-word recall), orientation (date, month, year, and day of the week; naming the President and Vice President), and executive function (clock drawing test). A possible dementia classification was assigned when the person scored ≤1.5 SD below the mean in one domain of the cognitive test. A probable dementia classification was assigned when the person was diagnosed with dementia or scored ≤1.5 standard deviations (SD) below the mean in at least two domains of the cognitive tests. Thus, possible dementia is a classification of less severe cognitive impairment than probable dementia. The score cut points for ≤1.5 SDs below the mean on NHATS cognitive domains were ≤3 for memory (score range 0 to 20), ≤3 for orientation (score range 0 to 8), and ≤1 for executive function (score range 0 to 5) (Kasper et al., 2023, 2024).

The NHATS’ broad definition of possible and probable dementia demonstrates high sensitivity compared to the “gold standard” ADAMS (Aging, Demographics, and Memory Study) dementia diagnostic criteria and reasonably good sensitivity against diagnoses of dementia or cognitive impairment not dementia (Kasper et al., 2023). Chyr et al. (2024) also found that the agreement between the subjective cognitive impairment report and the NHATS’ probable dementia was 90% and of substantial strength (prevalence- and bias-adjusted kappa, 0.80), with higher agreement rates in older and less-educated subgroups.

*Depression/anxiety symptoms in the past month in 2022 and 2023*: In NHATS, depression/anxiety symptoms were assessed with the Patient Health Questionnaire-4 (PHQ-4) (Kroenke et al., 2009). The PHQ-4 includes the first two items (PHQ-2; had little interest or pleasure in doing things, and felt down, depressed, or hopeless) from the 9-item PHQ-9 for depression (Kroenke et al., 2003) and the first two items (GAD-2; felt nervous, anxious, or on edge, and have been unable to stop or control worrying) from the 7-item Generalized Anxiety Disorder Scale (Spitzer et al., 2006). Responses to each PHQ-4 item were based on a 4-point scale (0 = not at all; 1 = several days; 2 = more than half the days; 3 = nearly every day), with the total score ranging from 0 to 12. The unweighted Cronbach’s alphas for the PHQ-4 for the study sample were 0.74 in 2022 and 0.77 in 2023. The PHQ-4 scores were also used to categorize symptom severity: no symptoms (0-2), mild symptoms (3-5), and moderate/severe symptoms (6-12) for descriptive purposes (Kroenke et al., 2009).

*Sociodemographic, health, and social support characteristics in 2023*: Demographic variables included age (65-74 [reference category], 75-84, 85+); gender (female vs. male [reference category]); race/ethnicity (non-Hispanic White [reference category], non-Hispanic Black, Hispanic, all other); and education (bachelor’s degree vs. no degree [reference category]). The health status variables included the number of chronic medical conditions (0-8: arthritis, cancer, hypertension, heart disease, stroke, diabetes, lung disease, osteoporosis), activity-limiting pain (yes vs. no), vision impairment (i.e., unable to see clearly across the street with or without glasses vs. able to see), and any hearing impairment (with or without hearing aids vs. no impairment). Social support was measured with the self-reported social support network size (0-5). The respondents were asked about up to five people they talk to about important things in life, including good or bad things that happen and problems or concerns they may have had. The 2024 Lancet Commission on Dementia Prevention and Intervention (Livingston et al., 2024) identified increased vision and hearing loss and social isolation as dementia risk factors.

### Analysis

All analyses were conducted with Stata/MP 19.5’s svy function (College Station, TX) to account for NHATS’s stratified, multistage sampling design (Freedman et al., 2020, 2024). First, we used χ^2^ and one-way ANOVA to compare demographic and health characteristics and depressive/anxiety symptoms by dementia status: No dementia, possible dementia, and probable dementia. We then used χ^2^ and *t* tests to compare the two dementia groups.

Second, to test cross-sectional, bidirectional associations between dementia status and depressive/anxiety symptoms in 2023 (H1), we first fitted a multinomial logistic regression model with dementia status (possible and probable dementia vs. no dementia) as the dependent variable and depressive/anxiety symptoms as the independent variable. We then fitted a linear regression model with depressive/anxiety symptoms as the dependent variable and dementia status as the independent variable. Age, gender, race/ethnicity, education, chronic medical conditions, pain, sensory impairments, and social support network size were entered as covariates. In the multinomial logistic regression model, we also controlled for the 2022 dementia status. In the linear regression model, we also controlled for 2022 depressive/anxiety symptoms. The multinomial logistic regression results are reported as RRRs with 95% confidence intervals (CIs). The linear regression results are reported as coefficients (B) and linearized standard errors (SE). Multicollinearity among covariates was not a concern based on a cut-off of 2.50 using the variance inflation factor test (Allison, 2012).

Third, to examine the one-year lagged associations between dementia status and depressive/anxiety symptoms (H2 & H3), we fitted a path model using generalized structural equation modeling (gsem in Stata) for a simultaneous estimation of multiple, potentially reciprocal relationships over time. The first and second path components tested the associations of the 2022 depressive/anxiety symptoms with the 2023 possible and probable dementia (H2), respectively, using a Bernoulli distribution and logit link, and including the 2022 dementia status, sociodemographic and health characteristics, and social support network size as covariates. The third path component tested the impact of 2022 possible and probable dementia on 2023 depressive/anxiety symptoms (H3), using a Gaussian distribution and identity link, and included 2022 depressive/anxiety symptoms, sociodemographic and health characteristics, and social support network size as covariates. The path model results are reported as coefficients (B) and linearized standard errors (SE). Statistical significance was set at *p* < .05.

## Results

### Demographic and Health Characteristics and Depressive/Anxiety Symptoms by Dementia Status

Table 1 shows that in 2023, 6.6% and 5.7% of the study sample were classified as having possible and probable dementia, respectively. Compared to those without dementia, those with possible or probable dementia were older and included a higher proportion of racial/ethnic minorities but a lower proportion of college graduates. In general, those with dementia had more health problems, higher depressive/anxiety symptoms, but a smaller social support network size. Of the two dementia groups, those with probable dementia were older and had higher depressive/anxiety symptoms, with approximately 22% of them showing moderate to severe symptom severity.

**Table 1.** Characteristics of older adults by dementia status in 2023

	All	No dementia (0)	Possible dementia (1)	Probable dementia (2)	*p* among all 3 groups	*p* between (1) & (2)
*N* (%)	4,942 (100)	4,102 (87.9)	447 (6.6)	393 (5.7)		
Age group (years, %)					<.001	.844
65-74	46.8	49.0	31.1	30.1		
75-84	40.9	40.9	42.0	40.5		
85+	12.3	10.1	26.9	29.4		
Female (%)	55.9	57.1	45.9	49.0	.001	.534
Race/ethnicity (%)					<.001	.051
Non-hispanic white	81.0	82.6	65.7	73.6		
Non-hispanic black	8.8	8.1	17.6	9.1		
Hispanic	5.4	4.7	10.1	10.4		
Other	4.9	4.6	6.6	6.9		
≥ Bachelor’s degree (%)	38.6	40.8	24.5	20.7	<.001	.344
No. Of chronic medical conditions, M (SE)	2.35 (0.03)	2.31 (0.03)	2.64 (0.09)	2.61 (0.13)	.003	.797
Activity-limiting pain (%)	32.2	31.8	34.6	35.5	.457	.864
Vision impairment (%)	5.5	4.5	12.6	12.3	<.001	.923
Hearing impairment (%)	10.1	8.9	15.2	23.5	<.001	.016
Social support network size, M (SE)	2.38 (0.04)	2.44 (0.05)	2.04 (0.08)	1.75 (0.07)	<.001	.006
Dementia status in 2022 (%)					<.001	<.001
No dementia	89.9	96.0	65.4	23.1		
Possible dementia	6.2	3.9	24.6	20.4		
Probable dementia	3.9	0.1	10.0	56.5		
Depressive/anxiety symptoms in 2022, M (SE)	1.63 (0.05)	1.53 (0.05)	2.39 (0.19)	2.67 (0.27)	<.001	<.001
Depressive/anxiety symptoms in 2023, M (SE)	1.64 (0.04)	1.51 (0.04)	2.08 (0.17)	3.01 (0.26)	<.001	.002
By symptom severity (%)					<.001	.003
Normal (0-2)	75.4	77.3	67.0	55.2		
Mild (3-5)	17.6	16.9	22.8	22.4		
Moderate/severe (6-12)	7.0	5.8	10.2	22.4		

*Note. p*-values are calculated based on Pearson’s χ^2^ tests for categorical variables and ANOVA and t-tests for continuous variables.

### Cross-Sectional Associations Between Dementia Status and Depressive/Anxiety Symptoms

The first two columns of Table 2 show that after adjusting for covariates, depressive/anxiety symptoms were associated with a higher likelihood of both possible and probable dementia compared to no dementia, with an even greater likelihood for probable dementia (RRR = 1.07, 95% CI = 1.01-1.14 for possible dementia; RRR = 1.20, 95% CI = 1.11-1.30 for probable dementia). The RRR for possible dementia fell outside the 95% CIs of probable dementia, indicating a significantly higher RRR for probable dementia relative to possible dementia. Among the covariates, the 2022 dementia status, especially probable dementia, yielded extremely large RRRs (e.g., RRR = 1574.81; 95% CI = 734.41-3376.90 for probable dementia). Even after adjusting for the 2022 dementia status, ages 85+ were associated with a higher likelihood of both possible and probable dementia. On the other hand, female gender, college education, and a larger social support network size were associated with a lower likelihood of both severity levels of dementia. Ages 75-84, being Black or Hispanic, and vision impairments were also associated with a higher likelihood of possible dementia.

**Table 2.** Cross-sectional associations between dementia and depressive/anxiety symptoms in 2023

	Dementia		Depressive/anxiety symptoms
	Possible dementia versus No dementia RRR (95% CI)	Probable dementia versus No dementia RRR (95% CI)	B (SE)
Those interviewed in both 2022 & 2023			
Depressive/anxiety symptoms in 2023	1.07 (1.01-1.14)*	1.20 (1.11-1.30)***	
Dementia in 2023: versus No dementia			
Possible dementia			0.19 (0.13)
Probable dementia			0.55 (0.20)*
Depressive/anxiety symptoms in 2022			0.58 (0.03)***
Dementia in 2022: versus No dementia			
Possible dementia	6.22 (4.12-9.37)***	13.59 (7.59-24.35)***	
Probable dementia	86.49 (37.68-198.53)***	1574.81 (734.41-3376.90)***	
Age group: versus 65-74 years			
75-84 years	1.62 (1.10-2.30)*	1.23 (0.69-2.18)	−0.22 (0.07)**
85+ years	3.60 (2.52-5.14)***	3.33 (1.89-5.86)***	−0.20 (0.08)*
Female versus Male	0.48 (0.35-0.66)***	0.42 (0.28-0.62)***	0.13 (0.06)
Race/ethnicity: versus Non-Hispanic white			
Non-hispanic black	2.24 (1.52-3.29)***	0.76 (0.42-1.36)	0.02 (0.09)
Hispanic	1.99 (1.27-3.12)**	1.33 (0.70-2.52)	0.03 (0.10)
Other	1.98 (1.02-3.83)*	1.97 (0.91-4.26)	0.13 (0.16)
College degree versus No degree	0.60 (0.43-0.83)**	0.46 (0.26-0.80)**	−0.20 (0.05)***
No. Of chronic medical conditions	1.07 (0.95-1.21)	1.01 (0.88-1.16)	0.10 (0.02)***
Activity-limiting pain versus No such pain	0.90 (0.63-1.29)	0.65 (0.40-1.04)	0.58 (0.09)***
Vision impairment versus No impairment	2.31 (1.44-3.69)**	1.54 (0.78-3.04)	0.63 (0.16)***
Hearing impairment versus No impairment	0.97 (0.70-1.36)	1.39 (0.81-2.39)	0.50 (0.14)**
Social support network size	0.89 (0.79-0.99)*	0.74 (0.63-0.86)***	0.01 (0.02)
Sample *N* = 4,942; population *N* = 24,655,918; design df = 56	F (30,27) = 59.04, p < .001		F (15,42) = 69.21; p < .001; *R*^*2*^ = 0.459

*p < .05; **p < .01; ***p < .001.

The last column of Table 2 presents the results of the linear regression analysis, where depressive/anxiety symptoms served as the dependent variable and dementia status as the independent variable. Only probable dementia (B [SE] = 0.55 [0.20]) was a significant factor. Among the covariates, 2022 depressive/anxiety symptoms, ages 75-84 and 85+ (compared to 65-74 years), and having a college degree were negatively associated with depressive/anxiety symptoms, whereas female gender, the number of chronic conditions, activity-limiting pain, and vision and hearing impairments were positively associated with depressive/anxiety symptoms.

### One-Year Lagged Associations Between Depressive/Anxiety Symptoms and Dementia

Table 3 shows that, controlling for 2022 possible and probable dementia and other covariates, 2022 depressive/anxiety symptoms were not significantly associated with 2023 possible dementia; however, they were significantly associated with 2023 probable dementia (B [SE] = 0.18 [0.03], *t* = 5.52, *p* < .001). Controlling for 2022 depressive/anxiety symptoms and other covariates, only probable dementia in 2022 was significantly associated with depressive/anxiety symptoms in 2023 (B [SE] = 0.47 [0.18], *t* = 2.64, *p* = .011).

**Table 3.** One-year cross-lagged associations between dementia and depressive/anxiety symptoms

	2023 possible dementia			2023 probable dementia			2023 depressive/anxiety symptoms		
	B (SE)	*t*-value	*p*	B (SE)	*t*-value	*p*	B (SE)	*t*-value	*p*
2022 possible dementia	1.55 (0.19)	8.08	<.001	2.31 (0.29)	8.02	<.001	0.34 (0.17)	1.92	.059
2022 probable dementia	0.90 (0.23)	3.87	<.001	5.53 (0.25)	22.10	<.001	0.47 (0.18)	2.64	.011
2022 depressive/anxiety symptoms	0.02 (0.03)	0.62	.535	0.18 (0.03)	5.52	<.001	0.58 (0.02)	23.31	<.001
Age 75-84	0.48 (0.17)	2.74	.008	0.12 (0.26)	0.47	.637	−0.22 (0.07)	−3.02	.004
Age 85+	1.12 (0.17)	6.69	<.001	0.86 (0.28)	3.10	.003	−0.20 (0.09)	−2.33	.023
Female	−0.60 (0.16)	−3.76	<.001	−0.71 (0.20)	−3.51	.001	0.11 (0.07)	1.69	.097
Non-hispanic black	0.84 (0.18)	4.57	<.001	−0.69 (0.32)	−2.15	.036	0.01 (0.09)	0.03	.978
Hispanic	0.62 (0.19)	3.19	.002	−0.01 (0.30)	−0.03	.978	0.02 (0.10)	0.19	.846
Other race	0.54 (0.32)	1.68	.098	0.60 (0.40)	1.52	.133	0.14 (0.16)	0.87	.390
College degree	−0.43 (0.16)	−2.76	.008	−0.66 (0.25)	−2.61	.011	−0.21 (0.05)	−4.11	<.001
No. Of chronic medical conditions	0.07 (0.06)	1.25	.216	−0.03 (0.07)	−0.46	.645	0.10 (0.02)	3.94	<.001
Activity-limiting pain	−0.01 (0.17)	−0.05	.963	−0.33 (0.26)	−1.24	.221	0.58 (0.09)	6.16	<.001
Vision impairment	0.80 (0.23)	3.52	.001	0.12 (0.34)	0.34	.734	0.64 (0.16)	3.99	<.001
Hearing impairment	−0.09 (0.16)	−0.56	.579	0.34 (0.27)	1.26	.213	0.49 (0.14)	3.45	.001
Social support network size	−0.09 (0.05)	−1.66	.103	−0.28 (0.07)	−3.91	<.001	0.01 (0.02)	0.05	.957

Sample *N* = 4,942; Population *N* = 24,655,918; Design df = 56.

## Discussion

In this study, we examined the bidirectional cross-sectional and one-year cross-lagged relationships between depressive/anxiety symptoms and possible and probable dementia among Medicare beneficiaries ages 65 and older who self-reported data related to both conditions. We were especially interested in the short-term temporal influence of dementia on depressive/anxiety symptoms and vice versa. Our findings show that in 2023, 12.3% of the study sample had possible or probable dementia, and about 8% experienced moderate to severe depressive/anxiety symptoms. Multivariable regression models demonstrated bidirectional cross-sectional associations, supporting the hypothesis that probable dementia would exhibit a stronger relationship with depressive/anxiety symptoms than possible dementia. This suggests a reciprocal, dose-response relationship in which more severe cognitive impairment is linked to more severe emotional distress, and vice versa. Importantly, this association held even after adjusting for baseline dementia status and depressive/anxiety symptoms, indicating that probable dementia exerts an independent influence on these symptoms over time.

In the path model examining one-year cross-lagged associations, the strongest predictor of 2023 dementia status was the 2022 dementia status, reflecting the largely irreversible diagnostic stability of dementia, especially probable dementia. After adjusting for baseline dementia status and other covariates, 2022 depressive/anxiety symptoms still showed a significant association with 2023 probable dementia, but not with possible dementia. This finding aligns with longer-term prior research suggesting that persistent depression and anxiety are important precursors to dementia onset and progression. However, the lack of a significant association between 2022 depressive/anxiety symptoms and 2023 possible dementia is noteworthy. One explanation is that a one-year window may be too short to detect lagged effects of depressive/anxiety symptoms on the early stages of cognitive decline, particularly when cognitive deficits are limited to a single domain (e.g., memory, orientation, or executive function). Despite this, our findings indicate that even within a short time frame, depressive/anxiety symptoms in the preceding year are significantly associated with subsequent probable dementia. A previous study showed that moderate to severe depression, along with older ages and specific cognitive deficits, predicted conversion from mild cognitive impairment to AD-type dementia, with an estimated one-year conversion rate of 10.1% (Defrancesco et al., 2017). An earlier one-year prospective study also found that both depression and anxiety significantly influenced incident cognitive impairment, although depression and anxiety had different associations according to sex and the nature (general and amnestic or non-amnestic) of cognitive impairment (Potvin et al., 2011).

Our findings also indicate that, after accounting for baseline depressive/anxiety symptoms in 2022, probable dementia, but not possible dementia, in 2022 was significantly associated with increased depressive/anxiety symptoms in 2023. This suggests that more severe cognitive impairment exerts an independent effect on the persistence or worsening of these mental health symptoms over time. Probable dementia likely introduces greater stressors, functional declines, worries about the future, and/or biological changes that contribute to depressive/anxiety symptoms. These symptoms are likely exacerbated not only by the direct impact of progressive cognitive and functional impairments, but also by the emotional toll of losing autonomy, control, and social roles, as well as by feelings of negative self-perception and shame (Aldridge et al., 2019; Kłosińska & Leszko, 2024; Torossian, 2021). Notably, longitudinal studies of representative samples of older adults in the US have also found that purpose in life declines significantly with the emergence and progression of cognitive impairment (Mroz et al., 2024; Sutin et al., 2023). The lack of a similar association for possible dementia may reflect the more modest cognitive and functional declines in this group, which may not independently drive emotional distress to the same extent. Overall, these findings reinforce the idea that depressive/anxiety symptoms become particularly entrenched in the context of more advanced cognitive impairment, highlight the distinct vulnerability of individuals with probable dementia, and underscore the need for proactive mental health screening and tailored interventions in this population.

Overall, the cross-lagged associations in our analyses highlight the intertwined trajectories of cognitive and emotional health in older adults, with probable dementia and depressive/anxiety symptoms mutually reinforcing each other even within the short follow-up period. The fact that prior-year dementia status was the strongest predictor of current-year dementia, and similarly for depressive/anxiety symptoms, underscores the chronicity and stability of these interrelated conditions in later life. Importantly, even after accounting for these temporal stabilities and potential progression, our findings of bidirectional relationships—observed only for probable dementia—suggest dynamic feedback loops between more severe cognitive decline and emotional dysregulation. Pathophysiological links, including neuroinflammatory processes and shared neurobiological pathways, may act synergistically to accelerate neurodegeneration and exacerbate both depression and dementia (Hakim, 2022; Huang et al., 2024; Linnemann & Lang, 2020). Additionally, high social deprivation (measured by socioeconomic status indicators), as well as social isolation, loneliness, and low levels of social engagement and physical activity, are associated with elevated risks of both depression and dementia (Freak-Poli et al., 2022; Gallagher et al., 2016; Hofbauer & Rodriguez, 2023; Livingston et al., 2024; Ni et al., 2024; Oken et al., 2024; Shen et al., 2022; Sutin et al., 2020).

Our findings on age, education, and race/ethnicity align with previous research. Increasing age is associated with a higher prevalence and incidence of dementia (Fiest et al., 2016) but a lower prevalence of depression/anxiety (Fields et al., 2022). The link between higher early-life educational attainment and higher cognitive levels, as well as slower cognitive decline, in later life has been well-established (Meng & D'Arcy, 2012; Zahodne et al., 2015). Prior education and financial resources at the time of disability onset have also been identified as protective factors against late-life depression (McGiffin et al., 2019). Besides lower education, lower income, and economic instability, the disproportionate burden of AD and other dementias among Black and Hispanic older adults is attributed to structural racism, perceived discrimination, disparities in health care access and quality, and poor neighborhood and built environment (Palms et al., 2025; Zahodne et al., 2019, 2020, 2021). While our findings showed female gender to be associated with a lower likelihood of dementia, this may be due to the selection of self-respondents only. A meta-analysis found AD prevalence is nearly twice as high in females, but vascular dementia is nearly twice as high in males (Cao et al., 2020). The inverse association between social network size and dementia is consistent with prior research (Arshad et al., 2025).

Our study shows that the number of chronic medical conditions and activity-limiting pain are significantly associated with depressive/anxiety symptoms, but they were not associated with possible or probable dementia. Given the significant impact of chronic medical conditions on depressive/anxiety symptoms, these health problems may have an indirect effect on dementia through depressive/anxiety symptoms. This is supported by the finding that the co-existence of diabetes and elevated depressive symptoms significantly accelerates cognitive decline over time, especially among those aged 50-64 years (Demakakos et al., 2017). Vision impairment remained significantly associated with possible dementia even after adjusting for baseline dementia status. As modifiable factors for dementia prevention (Livingston et al., 2024), sensory impairments deserve greater attention in older adults through targeted prevention and timely treatment strategies.

### Limitations

Our study has some limitations. First, the sample was restricted to self-respondents, thereby excluding older adults with more advanced stages of dementia. The exclusion was justified in this study; however, our findings may not be generalizable to those with more advanced dementia. Second, depressive/anxiety symptoms in the NHATS were measured with four items, although they had acceptable Cronbach’s alphas in both years. Self-reports of depressive/anxiety may also have been subjected to recall and social desirability bias, especially among those with advanced dementia. Third, as the observation was limited to 2 years, the trajectories of depression/anxiety and cognitive decline could not be examined. Previous research has shown that diverse depression trajectories have different impacts on cognitive function (Forbes et al., 2024). Fourth, as aforementioned, the one-year lag may also have been insufficient to detect the potential impact of mild depressive/anxiety symptoms on early cognitive decline, which may partly explain the nonsignificant cross-lagged association with possible dementia. A longer follow-up interval (e.g., 2 years) was not examined due to the design of our analytic sample and the need to preserve sample size and temporal alignment across key variables in the NHATS data. Fifth, only correlations, not causations, can be derived from cross-sectional survey data.

## Conclusions

Despite these limitations, our study provides empirical evidence that older adults commonly experience elevated depressive/anxiety symptoms in the context of cognitive decline. As individuals become increasingly aware of their deteriorating cognitive functioning and its impact on daily life, they may experience emotional distress, including sadness, hopelessness, fear, and heightened anxiety. These affective responses likely reflect an adaptive reaction to perceived functional losses, but the symptoms may evolve into clinically significant psychiatric symptoms. Importantly, our findings also indicate that baseline depressive/anxiety symptoms are significantly associated with probable dementia 1 year later, even after controlling for baseline dementia status. While causality cannot be established with observational survey data, these bidirectional relationships support the conceptualization of depressive/anxiety symptoms as both risk factors for and early clinical indicators of dementia. Our findings add to the growing body of evidence based on longer-term studies that depressive/anxiety symptoms may serve both as modifiable risk factors for dementia and as prodromal or early clinical indicators of cognitive impairment.

## Implications

Our findings underscore the importance of early identification and concurrent management of both cognitive decline and depressive/anxiety symptoms in older adults. Older adults experiencing a decline in cognitive function need timely support with emotional and coping skills to address depressive/anxiety symptoms, mitigate feelings of shame, and reduce the risk of social withdrawal. While evidence-based treatments for late-life depression and anxiety are available for older adults without cognitive impairment, more research is needed for psychosocial treatments for older adults experiencing cognitive decline and its negative impacts, including the loss of function and social roles, social exclusion, and depressive/anxiety symptoms in particular (Kishita et al., 2020; McDermott et al., 2019; Noone et al., 2019). Interventions targeting depression/anxiety in the early stages of cognitive impairment may help delay dementia progression. Similarly, emotional and social support, cognitive support, and rehabilitation strategies may reduce the emotional burden in those with more advanced dementia. Our findings also underscore the importance of holistic approaches that address physical, sensory, cognitive, and emotional health while accounting for social determinants to reduce the risk and burden of these interrelated conditions in later life. Future research should prioritize the development and evaluation of multidimensional interventions that target these interrelated pathways, including strategies to mitigate social deprivation, foster social engagement, and enhance resilience, ultimately reducing the burden of cognitive and emotional decline in later life.

## Research Ethics

The University of Texas at Austin’s Institutional Review Board exempted this study, a secondary analysis of de-identified public-domain data.

## Data Availability

The data used in this study (The National Aging and Health Trends Study) are in the public domain.*
